# Impact of MAFLD on HBV-Related Stage 0/A Hepatocellular Carcinoma after Curative Resection

**DOI:** 10.3390/jpm11080684

**Published:** 2021-07-21

**Authors:** Yen-Po Lin, Shu-Hsien Lin, Chih-Chi Wang, Chih-Che Lin, Ding-Wei Chen, Ching-Hui Chuang, Pao-Yuan Huang, Chao-Hung Hung, Shih-Yu Yang, Wei-Ru Cho, Yu-Syuan Chen, Ming-Chao Tsai

**Affiliations:** 1 School of medicine, Chung-Shan Medical University, Taichung 40201, Taiwan; youcryforlol@gmail.com (Y.-P.L.); dante44442@gmail.com (Y.-S.C.); 2 Division of Hepato-Gastroenterology, Department of Internal Medicine, Kaohsiung Chang Gung Memorial Hospital and Chang Gung University College of Medicine, Kaohsiung 83301, Taiwan; linsusan77628@gmail.com (S.-H.L.); paoyuan813@gmail.com (P.-Y.H.); chh4366@yahoo.com.tw (C.-H.H.); three0310@gmail.com (S.-Y.Y.); cavaliemirer@gmail.com (W.-R.C.); 3 Liver Transplantation Center and Department of Surgery, Kaohsiung Chang Gung Memorial Hospital and Chang Gung University College of Medicine, Kaohsiung 83301, Taiwan; ufel4996@gmail.com (C.-C.W.); immunologylin@gmail.com (C.-C.L.); 4 Center for Translational Research in Biomedical Sciences, Liver Transplantation Program and Department of Surgery, Kaohsiung Chang Gung Memorial Hospital and Chang Gung University College of Medicine, Kaohsiung 83301, Taiwan; dennis8870@gmail.com; 5 Department of Nursing, Meiho University, Pingtung 91202, Taiwan; Helen.ch.chuang@gmail.com; 6 Graduate Institute of Clinical Medical Sciences, Chang Gung University College of Medicine, Kaohsiung 83301, Taiwan

**Keywords:** metabolic-associated fatty liver disease (MAFLD), non-alcoholic fatty liver disease (NAFLD), chronic hepatitis B, hepato-cellular carcinoma, lean MAFLD

## Abstract

Backgrounds and Aim: Metabolic-associated fatty liver dis-ease (MAFLD) is a novel term proposed in 2020 to avoid the exclusion of certain subpopulations, though the application of this term in the real world is very limited. Here, we aimed to evaluate the impact of MAFLD on hepatitis B virus (HBV)-related hepatocellular carcinoma (HCC) after curative resection. Methods: Patients with chronic hepatitis B (CHB)-related HCC who received hepatectomy between January 2010 and December 2019 were consecutively selected. The association between histologically proven concurrent MAFLD and clinical outcomes were retrospectively analyzed. Results: Among the 812 eligible patients with CHB-related HCC, 369 (45.4%) were diagnosed with concurrent MAFLD. After a mean follow-up of 65 months, 303 patients (37.3%) developed HCC recurrence, 111 (13.7%) died, and 12 (1.5%) received liver transplantation. Although no differences in the incidences of HCC recurrence (HR: 0.902, 95% CI: 0.719–1.131, *p* = 0.370) and death or liver transplantation (HR: 0.743, 95% CI: 0.518–1.006, *p* = 0.107) were observed between patients with and without MAFLD in multivariate analysis, the patients with MAFLD tended to achieve better recurrent-free survival compared to patients without MAFLD. Notably, lean MAFLD (BMI < 23 kg/m^2^) was a relative risk factor for tumor recurrence (HR: 2.030, 95% CI: 1.117–3.690, *p* = 0.020) among patients with MAFLD. Conclusions: The overall prognosis in HBV-related early-stage HCC, in terms of HCC recurrence and death or liver transplantation, was not significantly different between patients with and without MAFLD. Among patients with MALFD, lean-MAFLD was a risk factor for HCC recurrence. Further studies are warranted to validate these results.

## 1. Introduction

Hepatocellular carcinoma (HCC) is the sixth most commonly diagnosed cancer worldwide and ranks fourth in terms of cancer mortality [1,2]. The high incidence of HCC in Asia compared to other regions of the world is related to the predominance of chronic hepatitis B (CHB) [3]. Although a number of therapeutic options exist, including liver transplantation, hepatectomy, and ablation, overall survival is still poor due to the high rate of recurrence [4]. Surgical resection is a potentially curative treatment for HCC, though the cumulative rates of recurrence remain high (50–60%) [5,6,7,8]. Several factors are prognostic for recurrence after HCC resection, including tumor size and differentiation, serum α-fetoprotein (AFP), microvascular invasion, cirrhosis, surgical margin, and metabolic syndrome [5,9,10].

Over the past four decades, non-alcoholic fatty liver dis-ease (NAFLD) has become the most prevalent chronic liver disease worldwide, in line with the increased prevalence of the features of metabolic syndrome [11,12]. The association be-tween non-alcoholic fatty liver disease (NAFLD) and HCC was well established recently [13]. In the USA, the proportion of individuals with non-alcoholic steatohepatitis (NASH) among candidates for liver transplantation for HCC increased 7.7-fold between 2002 and 2016 [14]. Considering that CHB is endemic in the Asia-Pacific region, the prevalence of concurrent NAFLD in HBV-related HCC is expected to increase. Unfortunately, previous international guidelines defined NAFLD by excluding a secondary cause of hepatis steatosis, including significant alcohol consumption, HBV infection, or other causes [15,16], which means NAFLD and CHB cannot coexist concurrently. Hence, data regarding the impact of NAFLD on HBV-related HCC is scarce.

In 2020, a panel of experts proposed changing the terminology from NAFLD to metabolic-associated fatty liver disease (MAFLD) [17,18]. The diagnosis of MAFLD is based on the presence of liver steatosis in addition to overweight/obesity, type 2 diabetes mellitus (T2DM), or metabolic dysregulation with at least two risk features including an increased waist circumference, pre-diabetes, hypertension, hypertriglyceridemia, and low serum high-density lipoprotein (HDL)-cholesterol levels [17,18]. Accordingly, MAFLD is likely to be more strongly associated with metabolic dysregulation related events than NAFLD. Most importantly, based on this new definition, both MAFLD and CHB can be diagnosed concurrently, whereas the criteria for NAFLD exclude viral hepatitis. Although MAFLD is reported to better identify patients with hepatic steatosis and metabolic disease than NAFLD [19], to date, there is very limited information about the application of this new terminology in the real world, especially in HBV endemic countries with a high prevalence of HCC. Indeed, whether co-existence of MAFLD influences the pathological characteristics and outcomes of HBV-related HCC has never been reported in the literature.

Therefore, in this study, we aimed to evaluate the clinicopathologic characteristics and outcomes of HBV-related HCC after curative liver resection (LR) among patients with and without MAFLD.

## 2. Methods

### 2.1. Study Design and Ethics

This study was designed as a multicenter cross-sectional retrospective study in Taiwan. The Institutional Review Board of Kaohsiung Chang Gung Memorial Hospital approved this study (IRB number: 201701632A3), with a waiver of the requirement for informed consent owing to the retrospective design of the study with minimal risk to the participants.

### 2.2. Study Population

The data were obtained from the Chang Gung Research Database (CGRD), which is derived from the largest private hospital system in Taiwan, Chang Gung Memorial Hospital (CGMH); the database is systematically updated annually to include new data generated at CGMH. The CGRD data is obtained from two medical centers and two regional hospitals: Keelung, Linkou, Chiayi, and Kaohsiung CGMH. We retrospectively reviewed the CGRD database and retrieved data for patients with HCC treated between January 2010 to December 2019.

The inclusion criteria were as follows: (1) patients diagnosed with only HBV infection based on the presence of hepatitis B surface and negative hepatitis C antibody; (2) Barcelona Clinic Liver Cancer (BCLC) stage 0 and A (early-stage) HCC; (3) who received curative liver resection and for whom a pathologic hepatic steatosis report was available.

### 2.3. Data Collection

All data were collected retrospectively from medical record at the time of surgery, including age, gender, presence of T2DM, hypertension, alcohol consumption, smoking history, serum biochemistry, and hepatitis B markers, and HBV DNA (detection limit of 20 IU/mL, Roche COBAS TaqMan; Roche Molecular System, Branchburg, NJ, USA). The histological features of the resected tumor, including satellite nodules, capsule invasion, microvascular invasion, tumor differentiation, histologic grade, and the cirrhosis were recorded.

### 2.4. Study Outcomes

The primary outcome was recurrence-free survival (RFS), defined as the interval between surgery and the date of diagnosis of the first HCC recurrence. The secondary outcome was overall survival (OS), defined as the interval between the date of surgery and death or liver transplantation, or date of last follow-up. The end date of follow-up was 31 December 2020.

### 2.5. Definition

The proposed criteria for diagnosis of MAFLD are based on evidence of hepatic steatosis (>5%) plus one of the following three criteria, namely overweight/obesity (BMI ≥ 23 kg/m^2^ in Asians), presence of type 2 diabetes mellitus (T2DM), or evidence of metabolic dysregulation (≥2 of the following metabolic risk abnormalities: waist circumference ≥ 90/80 cm in Asian men and women, blood pressure ≥ 130/85 mm Hg or specific drug treatment, plasma triglycerides ≥ 150 mg/dL or specific drug treatment, plasma high-density lipoprotein-cholesterol < 40 mg/dL for men and <50 mg/dL for women or specific drug treatment, prediabetes, a homeostasis model assessment of insulin resistance score (HOMA-IR index) ≥ 2.5, and a plasma high-sensitivity C-reactive protein (hs-CRP) level > 2 mg/L) [17].

The diagnosis of HCC was defined according the histopathology reports for surgically resected tumor tissues and based on the criteria of the practice guidelines of the EASL or AASLD [20,21]. HCC was staged according to the BCLC guide-lines [22]. Histologic grade of tumor differentiation was scored using the modified nuclear grading scheme outlined by the Edmondson and Steiner, with tumor grade categorized as well, moderately, and poorly differentiated [20]. Liver cirrhosis was defined as Ishak fibrosis score 5–6 from non-tumor part [21]. T2DM was defined based on the World Health Organization (WHO) National diabetic group criteria [23]. Lean-MAFLD was subclassified as a BMI < 23 kg/m^2^ in Asians [24].

### 2.6. Statistical Analysis

Statistical analyses were performed using SPSS Version 23.0. (IBM Corp., Armonk, NY, USA) for Windows. Continuous variables were expressed as means ± standard deviations, while categorical variables were summarized as frequencies and relative percentages. The relationship between RFS and OS was analyzed using Kaplan–Meier survival curves, and comparisons were determined using the log-rank test. Cox proportional hazards regression models was employed for univariate and multivariate analysis of the hazard ratio (HR) of RFS and OS. *p*-values < 0.05 were considered statistically significant.

## 3. Results

### 3.1. Patient Characteristics

**Figure 1:**
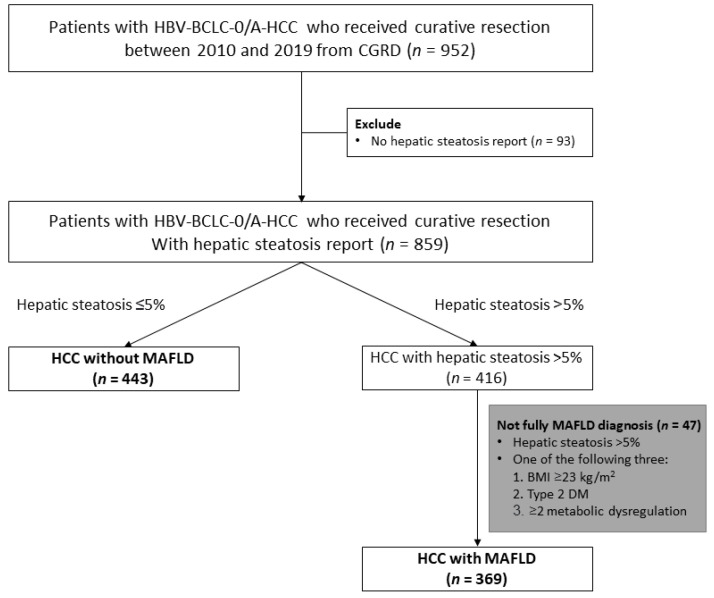
Patient selection flow diagram. Bold font is eligible for this analysis.

A total of 952 patients with BCLC stage 0 or A HCC received primary curative hepatectomy between January 2010 and December 2019. We excluded 93 patients without pathological hepatic steatosis reports and 47 patients whose clinical profiles were not available or undetermined in terms of the diagnostic criteria for MAFLD. Ultimately, the remaining 812 patients were eligible for this analysis (Figure 1); 443 patients had HCC with MAFLD (the MAFLD group) and the other 369 patients had HCC without MAFLD (the non-MAFLD group).

Table 1 presents the baseline clinicopathological characteristics of the study cohort. The mean age of the study population was 56.2 years, and the majority of patients were male (*n* = 693, 85.3%). The median tumor diameter was 2.7 cm and all patients had BCLC stage 0 (*n* = 189, 23.3%) or A (*n* = 623, 76.7%) HCC. Moreover, 199 patients (24.6%) were diabetic before surgery, and 410 patients (50.5%) were diagnosed with cirrhosis. Com-pared with the non-MAFLD group, patients in the MAFLD group had a significantly higher body mass index (BMI; *p* < 0.001), more frequently had T2DM (*p* < 0.001), hypertension (*p* < 0.001), and used statins (*p* = 0.008), had higher serum ALT (*p* = 0.001) and a higher platelet count (*p* < 0.001), and were more likely to have well-differentiated histologic grade (*p* < 0.001), but were less likely to have microvascular invasion (*p* < 0.001) and had lower serum AFP (*p* = 0.006).

### 3.2. Impact of MAFLD on the Outcomes of HBV-HCC

**Figure 2:**
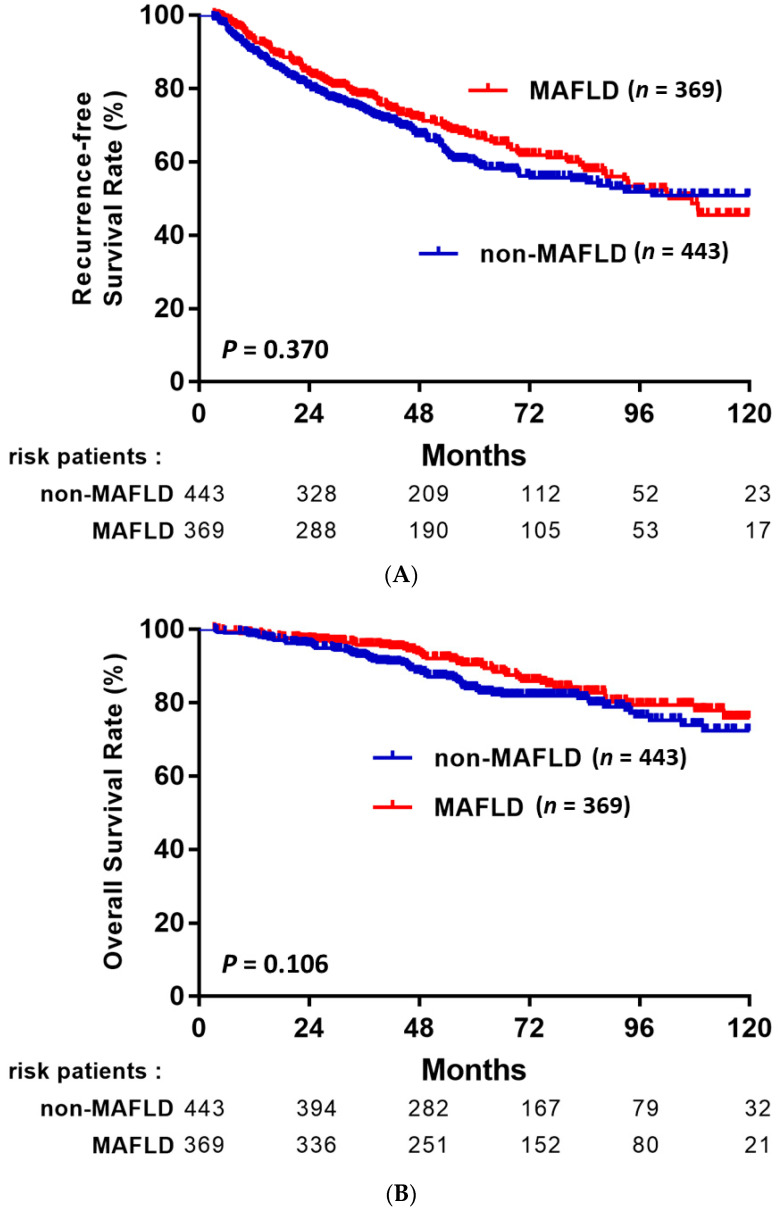
RFS (**A**) and OS (**B**) after curative resection in patients with HBV-related HCC with or without MAFLD.

**Figure 3:**
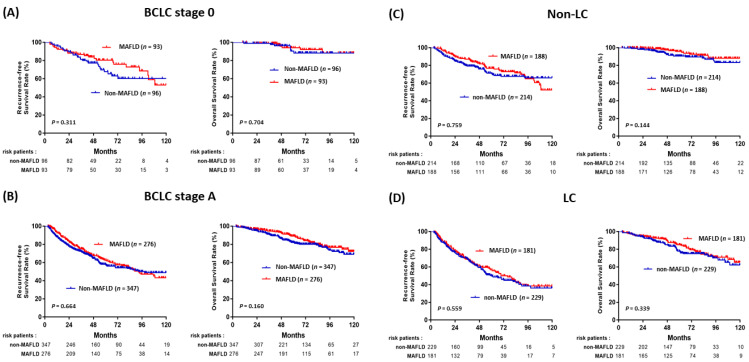
Comparisons of RFS and OS in HBV-HCC with or without MAFLD in the (**A**) BCLC stage 0, (**B**) BCLC stage A, (**C**) non-LC, and (**D**) LC subgroups.

After a mean follow-up of 65 months, 303 patients (37.3%) had developed recurrent HCC, 111 (13.7%) died, and 12 patients (1.5%) had received liver transplantation. The rates of RFS and OS after LR in HBV-related HCC among the patients with and without MAFLD are shown in Figure 2. Although there were no statistically significant differences between patients with MAFLD and without MAFLD, patients with MAFLD had favorable RFS and OS compared to patients without MAFLD (*p* = 0.37 and *p* = 0.106, respectively). In subgroup analysis based on various clinical characteristics (Figure 3). RFS and OS were not significantly different between the patients with MAFLD and without MAFLD in the BCLC stage 0 (Figure 3A), BCLC stage A (Figure 3B), no liver cirrhosis (Figure 3C), and liver cirrhosis (Figure 3D) subgroups.

### 3.3. Factors Associated with HCC Recurrence

The stepwise Cox proportional hazard model shown in Table 2 summarizes the prognostic factors associated with HCC recurrence in the study cohort. In this model, older age (hazard ratio [HR], 1.023; 95% CI, 1.003–1.042, *p* = 0.020) and liver cirrhosis (HR, 2.178; 95% CI, 1.146–3.282, *p* < 0.001) were related to a higher risk of recurrence; MAFLD was not associated with the risk of HCC recurrence (HR, 0.902; 95% CI, 0.719–1.131, *p* = 0.370); Albumin (hazard ratio [HR], 0.772; 95% CI, 0.569–0.915, *p* = 0.007) were related to a lower risk of recurrence.

### 3.4. Factors Associated with Overall Survival

As shown in Table 3, multivariate analysis revealed that current alcohol drinking (HR: 1.830, 95% CI: 1.032–3.244, *p* = 0.039), presence of cirrhosis (HR: 4.273, 95% CI: 1.934–9.439, *p* < 0.001), and a larger tumor size (HR: 1.515, 95% CI: 1.309–1.945, *p* < 0.001) were independent risk factors associated with death or liver transplantation. However, MAFLD was not significantly associated with overall survival (HR, 0.743; 95% CI, 0.518–1.006, *p* = 0.107).

### 3.5. MAFLD Subgroup Analysis: Lean-MAFLD Is a Risk Factor

**Figure 4:**
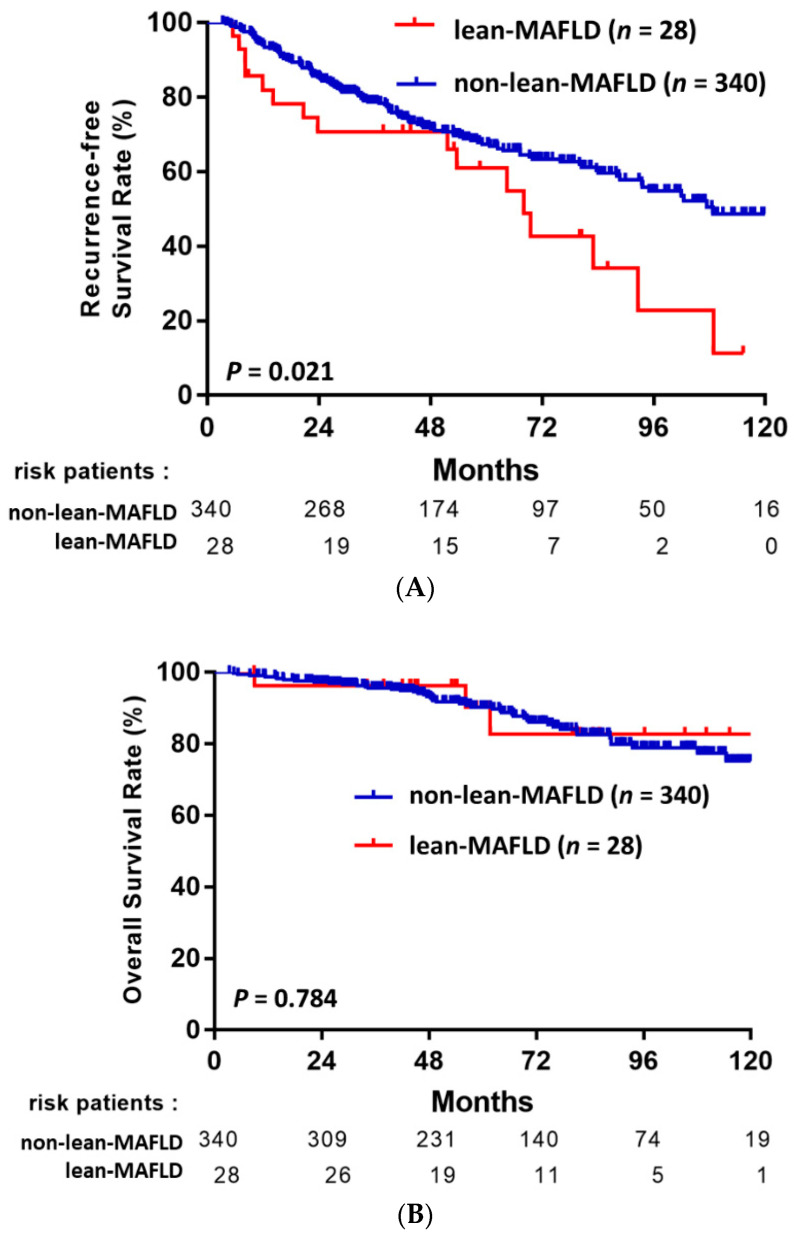
RFS (**A**) and OS (**B**) in HBV-MAFLD-HCC patients after curative resection stratified by the lean-MAFLD and non-lean-MAFLD subgroups.

NAFLD is increasingly being recognized in non-obese or lean individuals, especially in Asia, and lean individuals with NAFLD may even have poorer outcomes than obese individuals with NAFLD. To explore this issue, we further stratified our HCC-MAFLD cohort into a lean subgroup (BMI < 23 kg/m^2^) and non-lean subgroup (BMI ≥ 23 kg/m^2^). Among the 368 patients with MAFLD and HCC, 28 (7.6%) patients were classified as lean-MAFLD, and the other 340 were classified as non-lean-MAFLD. As shown in Figure 4A, patients with lean-MAFLD had significantly poorer RFS compared to patients with non-lean-MAFLD (*p* = 0.021). In contrast, the rate of death or liver transplantation did not significantly differ be-tween these two groups (*p* = 0.784, Figure 4B). In the multivariate analysis that included the entire MAFLD cohort of 369 patients, lean-MAFLD was associated with a significantly higher risk of HCC recurrence than non-lean MAFLD (HR, 2.030; 95% CI, 1.117–3.690, *p* = 0.020), independently of other predictive factors (Table 4). Other factors significantly associated with HCC recurrence were BCLC stage A (HR, 2.005; 95% CI, 1.238–3.247, *p* = 0.005), presence of cirrhosis (HR, 2.300; 95% CI, 1.572–3.366, *p* < 0.001), and presence of satellite nodules (HR, 4.239; 95% CI, 2.044–8.794, *p* < 0.001; Table 4).

The clinicopathological features of the patients with HCC in the lean-MAFLD (BMI < 23 kg/m^2^) and non-lean-MAFLD (BMI ≥ 23 kg/m^2^) subgroups are summarized in Table 5. Lean-MAFLD was associated with older age (*p* = 0.015), diabetes (*p* < 0.001), lower serum ALT (*p* = 0.048), and lower BMI (*p* < 0.001), but not with other characteristics, such as platelet count, serum AFP, microvascular invasion, or histological stage, that were significantly different between patients with and without MAFLD.

## 4. Discussion

This study aimed to evaluate the clinical impact of concurrent MAFLD on the prognosis of HCC patients with CHB. In this large multicenter study, we analyzed 812 consecutive patients who were classified into the MAFLD and non-MAFLD groups after curative resection for HBV-related early-stage HCC (BCLC stage 0 or A). The main finding was that MAFLD was not associated with either RFS or OS. Furthermore, we found that the lean-MAFLD subtype was an independent risk factor for RFS among HCC patients with MAFLD. To our knowledge, this is the first study to assess the associations between the new criteria for MAFLD and the outcomes of HBV-related HCC after curative resection.

Due to the increasing global prevalence of metabolic diseases, which may frequently co-exist with other etiologies that contribute to hepatic steatosis, the new concept of concomitant MAFLD and other liver diseases (dual etiology fatty liver disease) was first proposed in 2020 [17,18]. In contrast to NAFLD, for which the criteria are based on ‘negative’ assumptions, the newly proposed definition of MAFLD is based on ‘positive’ assumptions and emphasize the contribution of metabolic dysfunction. A recent review summarizing the newest studies that compared the clinical and prognostic characteristics of subjects with NAFLD and MAFLD was reported [25]. In Taiwan, Huang et al. examined and compared the clinical and histologic features of MAFLD versus NAFLD in patients with biopsy-proven hepatic steatosis. They reported that the novel diagnostic criteria for MAFLD include an additional 38.9% of patients with hepatic steatosis and help to better identify patients with a high degree of disease severity for early intervention than the previous NAFLD criteria [26]. A study conducted by Wang et al. in China in 2021 compared patients with MAFLD and HBV-MAFLD in a large biopsy proven cohort [27]. They reported that patients with HBV-MAFLD had similar metabolic features as patients with pure MAFLD, and the presence of HBV infection was associated with a lower grade of steatosis but higher grades of inflammation and fibrosis in MAFLD. Even though several studies have investigated and validated the clinical significance of the MAFLD criteria, huge controversies remain in regard to the change in definition from NAFLD to MAFLD for the description of fatty liver disease; notably, the consequences of this change on recurrence in HCC remain unclear.

In contrast to a previous study, our study was conducted in a HBV-endemic area and is the first evaluation of the impact of ‘pathologically-proven’ MAFLD on HBV-related HCC after curative resection. Hepatic steatosis can be detected using se-rum biomarkers, imaging techniques or histology; however, pathologic diagnosis remains the gold standard and is more reliable. We evaluated hepatic steatosis based on the pathological assessment of the resected non-tumor tissues, which is more accurate than evaluations of core biopsies, which was used from most published studies [26,27]. In the present study, we observed no significant differences between the RFS and OS of patients with and without MAFLD. This association remained consistent regardless of BCLC stage or the presence or absence of cirrhosis, which further confirms no significant differences among HCC patients with or without MAFLD. This result is similar, but not exactly the same, recently published Korean study [28]. Yoon et al. compared 196 patients with NAFLD and 142 without NAFLD, and concurrent NAFLD was not associated with either RFS and OS after resection in CHB-HCC [28]. In fact, according to the diagnostic criteria for NAFLD, CHB, and NAFLD cannot be concurrently diagnosed. Therefore, this unsynergistic result was unexpected. In fact, assessment of a large population cohort over 25 years revealed an increasing number of patients with HCC develop liver metabolic disorders, including NAFLD or MAFLD [29], and these diseases are emerging as new precancerous conditions in addition to the traditional well-characterized risk factor, viral-induced cirrhosis. Theoretically, we assumed patients with HBV-MAFLD-HCC would have poorer RFS and OS due to the ‘double-risk’ condition. In contrast, these patients seemed to have favorable RFS and OS compared to patients without MAFLD (Figure 2). Furthermore, the patients in the MAFLD group had a lower frequency of thrombocytopenia, lower serum AFP, a higher proportion of well differentiation tumors, and a lower proportion of microvascular invasion, which are so-called ‘positive’ factors for better outcomes in HCC. These pathological differences were also noted in the study by Yoon et al. [28], in which well-differentiated tumor stage and microvascular invasion were less frequent in the NAFLD group. Hence, we postulate that MAFLD exerts a protective effect against HCC recurrence in CHB-HCC. The possible reasons and relationships by which MAFLD affects tumor differentiation and vascular invasion remain still unclear. Further experimental studies are needed to explore the underlying mechanisms of HCC development in animal models of NAFLD/MAFLD.

We further stratified the patients with MAFLD into lean and non-lean-MAFLD subgroups. Notably, the lean-MAFLD patients had significantly poorer RFS than the non-lean-MAFLD patients (Figure 4). Currently, it is recognized that between 5% and 45% of patients with NAFLD in Asian populations are lean [30]. To the best of our knowledge, this is the first study to indicate the term ‘lean-MAFLD’ in the HCC study. The pathophysiology of lean MAFLD is puzzling and poorly understood. Lean-NAFLD patients are reported to have better metabolic and histologic profiles than individuals with obese-NAFLD, but may experience accelerated disease progression and poorer out-comes [31,32]. Genetic variations in PNPLA3, transmembrane 6 superfamily 2 (TM6SF2), membrane-bound O-acyltransferase domain-containing 7 (MBOAT7), or other genes associated with hepatic steatosis may possibly explain the relationship between lean NAFLD and the increased future risk of developing severe liver disease or RFS in HCC. In this study, there were no significant differences in any pathologic characteristics, including microvascular invasion or histological stage, between the lean- and non-lean-MAFLD groups; however, the lean-MAFLD group was older than the non-lean-MAFLD group, which was consistent with a recent study published from Taiwan in which the prevalence of lean MAFLD was higher in elder age [33], which may explain the higher risk of HCC recurrence in the lean-MAFLD group. Another possible reason may be that lean-MAFLD (BMI < 23 kg/m^2^) could be due to cancer-related malnutrition, which could also lead to poorer RFS in the lean-MAFLD group. In addition, the complex interaction among multiple factors including genetic, dietary, sugar intake, enterohepatic circulation, and gut microbiota is likely to modify individual metabolic health status between lean- and non-lean MAFLD patients. However, due to the retrospective design and the limited number of patients with lean-MAFLD in our study cohort, further studies of larger cohorts with comprehensive metabolic profiles, and gut microbiota analysis before and after HCC resection are required to evaluate the characteristics and prognosis of this special population.

This study has several strengths. Firstly, a large sample size of recently treated (from 2010 to 2019) patients was collected, which means the baseline characteristics and prevalence of metabolic disease are close to the rates of disease in the current population. Second, since referral is not required in Taiwan, there was no referral bias in the study. Even though some patients were lost to follow-up, we contacted these patients by phone and/or checked their vital status using the Cancer Screening and Tracing Information Integrated System for Taiwan. Therefore, we could confirm the vital status of each patient. Third, to reduce issues related to the heterogeneity of these diseases in Western populations (including environmental and genetic factors), we recruited a homogenous study population, which may help to draw more precise conclusions for Asian patients. Most importantly, this is the first study to investigate the im-pact of pathologically proven MAFLD on HBV-HCC after curative liver resection, which can provide more accurate steatosis evaluation than those by core biopsies or image.

Our approach has some inherent potential limitations. First of all, this was not a prospective study. However, we believe that the risk of bias was small, because most patients were followed by the same physicians throughout the course of their disease, with clinical and laboratory assessments and HCC screening using ultrasonography every 3–6 months. Second, we only enrolled patients with HBV-related early-stage HCC with or without MAFLD. Whether MAFLD has a similar impact on the prognosis of HCC caused by different etiologies (such as hepatitis C virus) or even advanced HCC remains to be investigated, and our results need to be validated in other cohorts and in Western countries. Third, not all data could be obtained from the electronic medical records, such as the HOMA-IR index, lipid profile, or waist circumference, resulting in the exclusion of some patients from the analysis, especially in the lean-MAFLD group. Future prospective studies of larger numbers of patients with HCC may help clarify this point.

## 5. Conclusions

In conclusion, the overall prognosis in terms of HCC recurrence and death or liver transplantation in CHB-related HCC were not significantly different between patients with and without MAFLD. However, concurrent MAFLD showed a mild survival ben-efit, which may be due to the higher frequency of well-differentiated tumors and lower frequency of microvascular invasion compared to patients without MAFLD. Moreover, patients in the lean-MAFLD group had a higher rate of tumor recurrence than patients with non-lean-MAFLD.

## Figures and Tables

**Table 1:** Characteristics of the 812 patients with HBV-related early-stage HCC with or without MAFLD who underwent curative resection.

	All Patients (*n* = 812)	HCC with MAFLD (*n* = 369)	HCC without MAFLD (*n* = 443)	*p*-Value
Age (years), mean ± SD	56.2 ± 10.7	56.2 ± 10.2	56.2 ± 11.1	0.312
Male gender, *n* (%)	693 (85.3)	320 (86.7)	373 (84.2)	0.908
Body mass index (kg/m^2^), mean ± SD	25.2 ± 3.2	26.7 ± 3.3	23.9 ± 3.4	<0.001
DM, *n* (%)	199 (24.6)	117 (31.7)	82 (18.6)	<0.001
Hypertension, *n* (%)	269 (33.1)	149 (40.4)	120 (27.1)	<0.001
Family history of HCC, *n* (%)	130 (16)	56 (15.2)	74 (16.7)	0.545
Alcohol drinking				0.744
Never, *n* (%)	596 (73.5)	273 (74.0)	323 (73.7)	
Current, *n* (%)	95 (11.7)	45 (12.2)	50 (11.3)	
Quit, *n* (%)	120 (14.8)	51 (13.8)	69 (15.6)	
Smoking				0.227
Never, *n* (%)	536 (66.1)	229 (64.8)	297 (67.2)	
Current, *n* (%)	160 (19.7)	82 (22.2)	78 (17.6)	
Quit, *n* (%)	115 (14.2)	48 (13.0)	67 (15.2)	
Platelets (<150 × 10^9^/L), *n* (%)	309 (39.4)	113 (31.3)	196 (46.3)	<0.001
AST (U/L), mean ± SD	36.8 ± 20.7	37.7 ± 20.6	36.0 ± 20.7	0.244
ALT (U/L), mean ± SD	42.5 ± 34.4	46.9 ± 37.8	38.9 ± 30.8	0.001
Total bilirubin (mg/dL), mean ± SD	0.8 ± 0.4	0.7 ± 0.4	0.8 ± 0.3	0.403
Albumin (g/dL), mean ± SD	4.2 ± 0.5	4.3 ± 0.5	4.2 ± 0.5	0.773
Creatinine (mg/dL), mean ± SD	1.1 ± 3.0	1.1 ± 4.1	1.1 ± 1.3	0.653
eGFR (mL/min/1.73^2^), mean ± SD	92.9 ± 24.4	94.2 ± 21.8	91.9 ± 26.4	0.185
AFP (>20 ng/mL), *n* (%)	344 (43.4)	138 (38.1)	206 (47.9)	0.006
AFP (>200 ng/mL), *n* (%)	170 (21.5)	62 (17.1)	108 (25.1)	0.006
Child–Pugh grade (A/B), *n* (%)	766/8 (99/1)	353/3 (99.2/0.8)	413/5 (98.8/1.2)	0.628
ALBI grade(I/II/III), *n* (%)	621/148/4 (80.3/19.1/0.6)	298/55/2 (83.9/15.5/0.6)	323/93/2 (77.3/22.2/0.5)	0.059
HBeAg positive, *n* (%)	83 (13.1)	33 (12)	50 (14)	0.428
HBV DNA				0.139
Undetectable, *n* (%)	146 (43.1)	66 (49.6)	80 (38.8)	
Detectable, *n* (%)	193 (56.9)	67 (50.4)	126 (61.2)	
NUCs treatment, *n* (%)	368 (45.3)	150 (40.7)	218 (49.2)	0.015
Ishak score, mean ± SD	4.1 ± 1.9	4.2 ± 1.7	4.0 ± 2.0	0.154
Liver cirrhosis, *n* (%)	410 (50.5)	181 (49.1)	229 (51.7)	0.453
BCLC stage 0/A, *n* (%)	189/623 (23.3/76.7)	93/276 (25.2/74.8)	96 / 347 (21.7/78.3)	0.236
Tumor size (cm) ^a^, mean ± SD	2.7 ± 1.0	2.7 ± 1.1	2.7 ± 1.0	0.695
Multiple tumors, *n* (%)	96 (11.8)	49 (13.3)	47 (10.6)	0.241
Histological grade				<0.001
Well-differentiated, *n* (%)	146 (18.0)	86 (23.4)	60 (13.6)	
Moderately differentiated, *n* (%)	511 (63.2)	191 (52)	320 (72.4)	
Poorly differentiated, *n* (%)	152 (18.8)	90 (24.5)	62 (14)	
Microvascular invasion, *n* (%)	250 (30.8)	90 (24.5)	160 (36.1)	<0.001
Capsule invasion, *n* (%)	654 (80.5)	296 (80.2)	358 (80.8)	0.543
Satellite nodules, *n* (%)	24 (3.0)	13 (3.6)	11 (2.5)	0.376
Follow-up (months), mean ± SD	65.1 ± 32.8	67.2 ± 32.6	63.4 ± 33.0	0.102

Data are expressed as mean ± standard deviation or *n* (%). ^a^ Diameter of the largest tumor nodule. Abbreviations: DM, diabetes mellitus; NUCs, nucleos(t)ide analogues; AST, aspartate aminotransferase; ALT, alanine aminotransferase; eGFR, estimated glomerular filtration rate; AFP, alpha fetoprotein; ALBI, albumin-bilirubin; HBV, hepatitis B virus; HBeAg, hepatitis B e antigen.

**Table 2:** Prognostic factors for HCC recurrence.

		Univariate		Multivariate	
Variable	Comparison	HR (95% CI)	*p*-Value	HR (95% CI)	*p*-Value
Age (years)	Per one-year increase	1.016 (1.006–1.027)	0.003	1.023 (1.003–1.042)	0.020
Sex	Male vs. Female	1.201 (0.858–1.908)	0.286		
DM	Yes vs. No	1.492 (1.166–1.908)	0.001		
Hypertension	Yes vs. No	1.148 (0.907–1.454)	0.251		
Alcohol drinking	Current vs. Never/Past	1.106 (0.863–1.418)	0.426		
Smoking	Current vs. Never/Past	1.168 (0.924–1.477)	0.195		
HCC family history	Yes vs. No	1.153 (0.864–1.539)	0. 332		
HBeAg	Positive vs. Negative	1.278 (0.889–1.839)	0.186		
HBV DNA (IU/mL)	Detectable vs. Undetectable	1.071 (0.737–1.555)	0.720		
NUCs treatment	Yes vs. No	1.056 (0.837–1.333)	0.664		
AST (U/L)	>40 vs. ≤40	1.462 (1.142–1.874)	0.003		
ALT (U/L)	>40 vs. ≤40	1.301 (1.029–1.647)	0.028		
Platelets (10^9^/L)	<150 vs. ≥150	1.524 (1.208–1.922)	<0.001		
AFP (ng/mL)	>200 vs. ≤200	1.028 (0.776–1.361)	0.847		
Albumin (mg/dL)	Per 1 unit decrease	1.295 (1.093–1.757)	0.007		
Child–Pugh class	B vs. A	1.209 (0.387–3.775)	0.744		
ALBI grade	II/III vs. I	1.166 (0.878–1.548)	0.289		
Liver cirrhosis	Yes vs. No	2.117 (1.673–2.679)	<0.001	2.178 (1.146–3.282)	<0.001
BCLC stage	A vs. 0	1.615 (1.195–2.182)	0.002		
Tumor no.	Multiple vs. Single	1.339 (0.985–1.819)	0.062		
Tumor diameter (cm)	Per 1 cm increase	1.218 (1.090–1.361)	<0.001		
Histological grade	Poor/Moderate vs. Well	1.230 (0.908–1.667)	0.181		
Microvascular invasion	Yes vs. No	1.150 (0.902–1.467)	0.258		
Capsule invasion	Yes vs. No	1.022 (0.766–1.363)	0.883		
Satellite nodules	Yes vs. No	2.418 (1.437–4.060)	0.001		
MAFLD	Yes vs. No	0.902 (0.719–1.131)	0.370		

Abbreviations: HR, hazard ratio; CI, confidence interval; BMI, body mass index; ETV, entecavir; TDF, tenofovir disoproxil fumarate; AST, aspartate aminotransferase; ALT, alanine aminotransferase; AFP, alpha fetoprotein; ALBI, albumin-bilirubin; HCC, hepatocellular carcinoma; HBeAg, hepatitis B e antigen; NUCs, nucleos(t)ide analogs.

**Table 3:** Prognostic factors associated with mortality/liver transplantation.

		Univariate		Multivariate	
Variable	Comparison	HR (95% CI)	*p*-Value	HR (95% CI)	*p*-Value
Age (years)	Per one-year increase	1.017 (1.000–1.034)	0.051		
Sex	Male vs Female	1.188 (0.702–2.009)	0.521		
DM	Yes vs. No	1.215 (0.815–1.812)	0.339		
Hypertension	Yes vs. No	1.183 (0.817–1.712)	0.374		
Alcohol drinking	Current vs. Never/Past	1.357 (0.934–1.971)	0.109	1.830 (1.032–3.244)	0.039
Smoking	Current vs. Never/Past	1.438 (1.004–2.059)	0.048		
HCC family history	Yes vs. No	0.840 (0.515–1.370)	0.485		
HBeAg	Positive vs. Negative	1.208 (0.685–2.128)	0.514		
HBV DNA (IU/mL)	Detectable vs. Undetectable	1.553 (0.903–2.670)	0.112		
NUCs treatment	Yes vs. No	0.852 (0.735–1.143)	0.452		
AST (U/L)	>40 vs. ≤40	1.417 (0.965–2.080)	0.075		
ALT (U/L)	>40 vs. ≤40	1.197 (0.825–1.736)	0.343		
Platelets (10^9^/L)	<150 vs. ≥150	2.096 (1.448–3.033)	<0.001		
AFP (ng/mL)	>200 vs. ≤200	1.003 (0.645–1.558)	0.990		
Albumin (mg/dL)	Per 1 unit decrease	1.536 (1.063–2.222)	0.023		
Child–Pugh class	B vs. A	3.332 (1.056–10.509)	0.040		
ALBI grade	II/III vs. I	1.286 (0.834–1.985)	0.255		
Liver cirrhosis	Yes vs. No	2.708 (1.823–4.023)	<0.001	4.273 (1.934–9.439)	<0.001
BCLC stage	A vs. 0	2.460 (1.384–4.373)	0.002		
Tumor no.	Multiple vs. Single	1.053 (0.638–1.738)	0.840		
Tumor diameter (cm)	Per 1 cm increase	1.458 (1.239–1.716)	<0.001	1.515 (1.309–1.945)	<0.001
Histological grade	Poor/Moderate vs Well	1.193 (0.739–1.926)	0.471		
Microvascular invasion	Yes vs. No	1.728 (1.204–2.479)	0.003		
Capsule invasion	Yes vs. No	1.362 (0.826–2.246)	0.226		
Satellite nodules	Yes vs. No	2.957 (1.442–6.062)	0.003		
MAFLD	Yes vs. No	0.743 (0.518–1.006)	0.107		

Abbreviations: HR, hazard ratio; CI, confidence interval; BMI, body mass index; ETV, entecavir; TDF, tenofovir disoproxil fumarate; AST, aspartate aminotransferase; ALT, alanine aminotransferase; AFP, alpha fetoprotein; ALBI, albumin-bilirubin; HCC, hepatocellular carcinoma; HBeAg, hepatitis B e antigen; NUCs, nucleos(t)ide analogs.

**Table 4:** Prognostic factors for HCC recurrence in the MAFLD group (*n* = 368).

		Univariate		Multivariate	
Variable	Comparison	HR (95% CI)	*p*-Value	HR (95% CI)	*p*-Value
Age(year)	Per one-year increase	1.007 (0.991–1.023)	0.402		
Sex	Male vs. Female	1.253 (0.741–2.118)	0.400		
DM	Yes vs. No	1.274 (0.894–1.816)	0.180		
Hypertension	Yes vs. No	1.167 (0.829–1.642)	0.377		
Alcohol drinking	Current vs. Never/Past	0.981 (0.667–1.443)	0.922		
Smoking	Current vs. Never/Past	1.256 (0.888–1.777)	0.197		
HCC family history	Yes vs. No	1.382 (0.906–2.109)	0. 134		
HBeAg	Positive vs. Negative	1.103 (0.603–2.015)	0.751		
HBV DNA (IU/mL)	Detectable vs. Undetectable	1.376 (0.747–2.536)	0.306		
NUCs treatment	Yes vs. No	1.091 (0.903–1.846)	0.662		
AST (U/L)	>40 vs. ≤40	1.522 (1.056–2.194)	0.024		
ALT (U/L)	>40 vs. ≤40	1.269 (0.896–1.798)	0.180		
Platelets (10^9^/L)	<150 vs. ≥150	1.314 (0.914–1.890)	0.140		
AFP (ng/mL)	>5 vs. ≤5	1.383 (0.954–2.004)	0.087		
Albumin (mg/dL)	Per 1 unit decrease	1.245 (0.862–1.799)	0.242		
Child–Pugh class	B vs. A	1.537 (0.214–11.031)	0.669		
ALBI grade	II/III vs. I	1.039 (0.650–1.662)	0.873		
Liver cirrhosis	Yes vs. No	2.168 (1.527–3.077)	<0.001	2.300 (1.572–3.366)	<0.001
BCLC stage	A vs. 0	1.777 (1.134–2.786)	0.012	2.005 (1.238–3.247)	0.005
Tumor no.	Multiple vs. Single	1.524 (1.004–2.314)	0.048		
Tumor diameter (cm)	Per 1 cm increase	1.145 (0.964–1.361)	0.123		
Histological grade	Poor/Moderate vs. Well	1.134 (0.757–1.698)	0.542		
Microvascular invasion	Yes vs. No	1.185 (0.802–1.751)	0.394		
Capsule invasion	Yes vs. No	1.069 (0.696–1.641)	0.760		
Satellite nodules	Yes vs. No	4.481 (2.337–8.592)	<0.001	4.239 (2.044–8.794)	<0.001
Lean-MAFLD	Yes vs. No	1.834 (1.087–3.095)	0.023	2.030 (1.117–3.690)	0.020

Abbreviations: HR, hazard ratio; CI, confidence interval; BMI, body mass index; ETV, entecavir; TDF, tenofovir disoproxil fumarate; AST, aspartate aminotransferase; ALT, alanine aminotransferase; AFP, alpha fetoprotein; ALBI, albumin-bilirubin; HCC, hepatocellular carcinoma; HBeAg, hepatitis B e antigen; NUCs, nucleos(t)ide analogs.

**Table 5:** Comparison of characteristics among patients with MAFLD-HCC stratified by lean (BMI < 23 kg/m^2^) and non-lean (BMI ≥ 23 kg/m^2^).

	Lean-MAFLD (*n* = 28)	Non-Lean-MAFLD (*n* = 340)	*p*-Value
Age (years), mean ± SD	60.7 ± 9.2	55.8 ± 10.2	0.015
Male gender, *n* (%)	21 (75)	299 (87.9)	0.051
Body mass index (kg/m^2^), mean ± SD	21.4 ± 1.4	27.1 ± 3.0	<0.001
DM, *n* (%)	19 (67.9)	98 (28.8)	<0.001
Hypertension, *n* (%)	14 (50)	135 (39.7)	0.286
Family history of HCC, *n* (%)	4 (14.3)	52 (15.3)	0.886
Platelets (<150 10^9^/L), *n* (%)	9 (33.3)	104 (31.2)	0.821
AST (U/L), mean ± SD	32.3 ± 15.0	38.2 ± 20.9	0.153
ALT (U/L), mean ± SD	33.2 ± 15.7	48.1 ± 38.9	0.048
Total bilirubin (mg/dL), mean ± SD	0.8 ± 0.4	0.7 ± 0.4	0.484
Albumin (g/dL), mean ± SD	4.1 ± 0.6	4.3 ± 0.4	0.162
Creatinine (mg/dL), mean ± SD	1.1 ± 4.1	1.1 ± 1.3	0.653
eGFR (ml/min/1.73^2^), mean ± SD	94.2 ± 21.8	91.9 ± 26.4	0.185
AFP (>20 ng/mL), *n* (%)	9 (33.3)	128 (38.3)	0.607
Child–Pugh grade (A/B)*, n* (%)	28/0 (100/0)	325/3 (99.1/0.9)	0.618
ALBI grade (I/II/III), *n* (%)	19/8/0 (70.4/29.6/0)	278/47/2 (85/14.4/0.6)	0.103
Liver cirrhosis, *n* (%)	10 (35.7)	171 (50.3)	0.138
BCLC stage 0/A, *n* (%)	5/23 (17.9/82.1)	87/253 (25.6/74.4)	0.364
Tumor diameter (cm), mean ± SD	2.6 ± 1.0	2.7 ± 1.1	0.768
Multiple tumors, *n* (%)	3 (10.7)	46 (13.5)	0.673
Histological grade			0.529
Well differentiated, *n* (%)	5 (18.5)	8 (23.6)	
Moderately differentiated, *n* (%)	13 (48.1)	178 (52.5)	
Poorly differentiated, *n* (%)	9 (33.3)	81 (23.9)	
Microvascular invasion, *n* (%)	5 (17.9)	85 (25.1)	0.394
Capsule invasion, *n* (%)	24 (85.7)	271 (79.9)	0.460
Satellite nodule, *n* (%)	13 (3.6)	11 (2.5)	0.376
Follow-up (months), mean ± SD	65.3 ± 30.4	67.2 ± 32.7	0.764
Recurrence, *n* (%)	16 (57.1)	119 (35.0)	0.019
Death or liver transplantation, *n* (%)	3 (10.7)	46 (13.5)	0.673

Data are expressed as mean ± standard deviation or *n* (%). Abbreviations: ETV, entecavir; TDF, tenofovir disoproxil fumarate; NUCs, nucleos(t)ide analogs; AST, aspartate aminotransferase; ALT, alanine aminotransferase; eGFR, estimated glomerular filtration rate; AFP, alpha fetoprotein; ALBI, albumin-bilirubin; HBV, hepatitis B virus; HBeAg, hepatitis B e antigen.

## Data Availability

All analyzed data are included in this published article. The original data are not suitable for publication owing to confidentiality issues, and only available upon rea-sonable request to the corresponding author.

## Abbreviations

MAFLD	metabolic-associated fatty liver disease
HCC	hepatocellular carcinoma
BCLC	Barcelona Clinic Liver Cancer
OS	overall survival
RFS	recurrence-free survival
HR	hazard ratio
HBV	Hepatitis B virus
AFP	alpha-fetoprotein
HBsAg	hepatitis B surface antigen
HBeAg	hepatitis B e antigen
BMI	body mass index
